# Conserved and non-conserved RNA–target modules in plants: lessons for a better understanding of *Marchantia* development

**DOI:** 10.1007/s11103-023-01392-y

**Published:** 2023-11-22

**Authors:** Halina Pietrykowska, Alisha Alisha, Bharti Aggarwal, Yuichiro Watanabe, Misato Ohtani, Artur Jarmolowski, Izabela Sierocka, Zofia Szweykowska-Kulinska

**Affiliations:** 1 Department of Gene Expression, Institute of Molecular Biology and Biotechnology, Faculty of Biology, Adam Mickiewicz University, Uniwersytetu Poznanskiego 6, 61-614 Poznan, Poland; 2 Department of Life Sciences, Graduate School of Arts and Sciences, The University of Tokyo, Tokyo, 153-8902 Japan; 3 Division of Biological Science, Graduate School of Science and Technology, Nara Institute of Science and Technology, Ikoma, 630-0192 Nara Japan; 4 Department of Integrated Biosciences, Graduate School of Frontier Sciences, The University of Tokyo, Kashiwa, 277-8562 Chiba Japan; 5 RIKEN Center for Sustainable Resource Science, Yokohama, 230-0045 Kanagawa Japan

**Keywords:** *Marchantia polymorpha*, *Physcomitrium patens*, *Arabidopsis thaliana*, MicroRNA, Long non-coding RNA, Development

## Abstract

A wide variety of functional regulatory non-coding RNAs (ncRNAs) have been identified as essential regulators of plant growth and development. Depending on their category, ncRNAs are not only involved in modulating target gene expression at the transcriptional and post-transcriptional levels but also are involved in processes like RNA splicing and RNA-directed DNA methylation. To fulfill their molecular roles properly, ncRNAs must be precisely processed by multiprotein complexes. In the case of small RNAs, DICER-LIKE (DCL) proteins play critical roles in the production of mature molecules. Land plant genomes contain at least four distinct classes of DCL family proteins (DCL1–DCL4), of which DCL1, DCL3 and DCL4 are also present in the genomes of bryophytes, indicating the early divergence of these genes. The liverwort *Marchantia polymorpha* has become an attractive model species for investigating the evolutionary history of regulatory ncRNAs and proteins that are responsible for ncRNA biogenesis. Recent studies on *Marchantia* have started to uncover the similarities and differences in ncRNA production and function between the basal lineage of bryophytes and other land plants. In this review, we summarize findings on the essential role of regulatory ncRNAs in *Marchantia* development. We provide a comprehensive overview of conserved ncRNA–target modules among *M. polymorpha*, the moss *Physcomitrium patens* and the dicot *Arabidopsis thaliana*, as well as *Marchantia*-specific modules. Based on functional studies and data from the literature, we propose new connections between regulatory pathways involved in *Marchantia*’s vegetative and reproductive development and emphasize the need for further functional studies to understand the molecular mechanisms that control ncRNA-directed developmental processes.

## Key message

We provide a comprehensive overview of conserved ncRNA–target modules amongst *Marchantia polymorpha*, *Physcomitrium patens* and *Arabidopsis thaliana* as well as  *Marchantia*-specific modules what contributes to a better understanding of the evolution of ncRNAs in terrestrial plants

## Introduction

In plants, regulatory non-coding RNAs (ncRNAs) are involved in diverse developmental processes, including shoot apical meristem (SAM) development, leaf morphogenesis, the juvenile-to-adult phase transition, determination of flowering time, fertilization and stress responses (Macintosh et al. 2001; Chen 2005; Mattick et al. 2009; Wang and Chekanova 2017; Hou et al. 2019). Non-coding RNAs are grouped into short (miRNA, siRNA and tasiRNA, among others) and long ncRNAs (Yu et al. 2019; Wierzbicki et al. 2021). MicroRNAs (miRNAs) are a class of short ncRNAs frequently 20–24 nt long that modulate gene expression through a mechanism of post-transcriptional gene silencing: mRNA cleavage or translational inhibition (Bartel 2004; Yu and Wang 2010; Bajczyk et al. 2023). Short interfering RNAs (siRNAs) represent a class of small, double-stranded RNAs (dsRNAs), usually 21–24 nt in length. These are derived from dsRNA precursors that are processed into siRNA duplexes by DICER-LIKE (DCL) activities. They suppress the expression of target mRNAs that correspond to dsRNA or are involved in the silencing and methylation of homologous DNA (Hamilton and Baulcombe 1999; Hamilton et al. 2002; Xie et al. 2004; Axtell 2013). Another class of small ncRNAs, trans-acting small interfering RNAs (tasiRNAs), are secondary siRNAs that silence their target RNAs in a similar manner as miRNAs, via mRNA cleavage or translational inhibition. In contrast, long non-coding RNAs (lncRNAs) are more than 200 nt in length and act in multiple ways to regulate their targets, resulting in chromatin remodeling, transcriptional enhancing or repressing, RNA splicing and target mimicry (F. Liu et al. 2010b; Wang and Chekanova 2017; Palos et al. 2023).

**Fig. 1:**
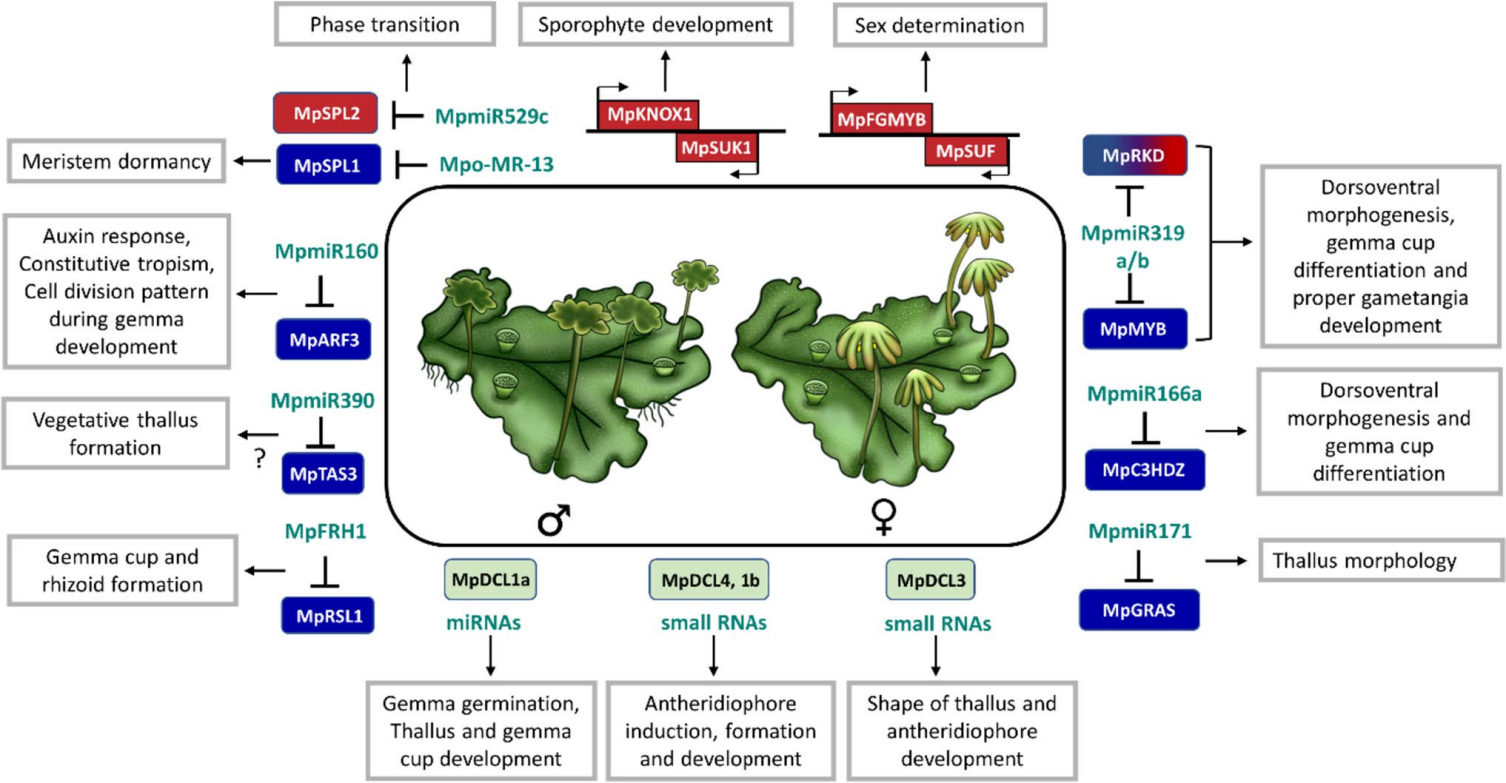
Schematic illustration of the role of miRNA–target modules that have been studied to date in *M. polymorpha* development. Modules that are involved in gametophyte development are shown in red, whereas modules involved in the vegetative development program are shown in blue. Since Mp*RKD* has roles in both vegetative and reproductive development, it is shown in a gradient of blue to red. DCLs, master producers of sRNAs, are shown in light green. Arrows and T-bars represent activation and repression, respectively. The question mark on the miR390–TAS3 module indicates that the exact role of this module needs to be determined in future functional studies. Abbreviations: miR, microRNA; ARF, AUXIN RESPONSIVE FACTOR; SPL, SQUAMOSA PROMOTER BINDING PROTEIN-LIKE; KNOX, KNOTTED1-like Homeobox; SUK, SUPPRESSOR of KNOX1; FGMYB, FEMALE GAMETOPHYTE MYB; SUF, SUPPRESSOR OF FEMINIZATION; C3HDZ, C-III HOMEODOMAIN-LEUCINE ZIPPER; DCL, DICER-LIKE; RSL1, ROOT HAIR DEFECTIVE SIX-LIKE 1; FRH1, FEW RHIZOIDS1; RKD, RWP-RK DOMAIN-CONTAINING (Tsuzuki et al. 2016, 2019; Flores-Sandoval et al. 2018; Lin and Bowman 2018; Hisanaga et al. 2019a; Thamm et al. 2020; Dierschke et al. 2021; Csicsely et al. 2023; Futagami et al. 2023; Streubel et al. 2023; Yelina et al. 2023)

Bryophytes, including liverworts, hornworts and mosses, originated from a group of early diverged land plant lineages (Qiu and Palmer 1999; Shaw et al. 2011; Morris et al. 2018; Donoghue et al. 2021). *Marchantia polymorpha* (*Marchantia*) is a complex thalloid liverwort that has gained much attention in recent years as a model system because of its use in advancing genetic and functional research (Ishizaki et al. 2016; Shimamura 2016; Bowman et al. 2017; Bowman 2022). The moss *Physcomitrium patens* (*Physcomitrium*) is another model organism used for studies on plant evolution, development, metabolism and genetics (Reski et al. 1994; Cove et al. 2006; Rensing et al. 2008; Prigge and Bezanilla 2010; Falz and Müller-Schüssele 2019). Although most of the knowledge about ncRNAs has arisen from angiosperms, the availability of several genome sequences, along with the use of high-throughput RNA sequencing, has led to initial insights into small RNA and lncRNA machineries in bryophytes (Arif et al. 2012; Alaba et al. 2015; Coruh et al. 2015; Lin et al. 2016; Tsuzuki et al. 2016; Lin and Bowman 2018; Perroud et al. 2018; Meyberg et al. 2020). Research on ncRNAs in *Marchantia* and *Physcomitrium* revealed their significant roles in different molecular mechanisms governing the development of these bryophytes and their responses to environmental cues. Functional analysis of miRNAs and their targets in angiosperms and bryophytes indicates that some developmental pathways are regulated by the same conserved modules across all land plants. However, some miRNA–target modules diversified in function during the course of evolution. The presence of various conserved and *Marchantia*-specific gene regulatory modules in which ncRNAs are involved provides new insights leading to a better understanding of *Marchantia* development (Fig. 1). In this review, we summarize the essential role of regulatory ncRNAs in *Marchantia* and propose new putative connections between already known modules that are crucial for life cycle completion in *Marchantia*.

## DCLs, producers of small RNAs

DCL proteins have the critical function of producing small RNAs from long dsRNA precursors. *Arabidopsis thaliana* (*Arabidopsis*) has four DCL proteins (AtDCL1–AtDCL4), and their molecular functions have been characterized by genetic studies (Bologna and Voinnet 2014) as well as biochemical assays (Kurihara and Watanabe 2004; Fukudome et al. 2011; Nagano et al. 2014). Now we know that AtDCL1 is involved in miRNA biogenesis from primary microRNA (pri-miRNA) transcripts (Park et al. 2002; Kurihara and Watanabe 2004), and the other proteins, AtDCL2–AtDCL4, function in the biogenesis of siRNAs with specific sizes of 22, 24 and 21 nt, respectively (Xie et al. 2004; Akbergenov et al. 2006; Henderson et al. 2006). Reflecting the importance of miRNAs for plant development, AtDCL1 knockout mutants are embryonic lethal (Schauer et al. 2002). Notably, 24 nt siRNAs (heterochromatic siRNAs, hc-siRNAs) are produced by AtDCL3 from precursors generated by RNA polymerase IV from heterochromatic genomic regions. They are involved in transcriptional gene silencing (TGS) via RNA-directed DNA methylation of transposable elements. In contrast, 21 nt siRNAs produced by AtDCL4 are important for post-transcriptional gene silencing (PTGS) or RNA interference to regulate endogenous gene expression. Moreover, critical targets of AtDCL4 include phased tasiRNAs derived from the *TAS* gene loci, including *TAS3* (Xie et al. 2005; Yoshikawa et al. 2005). The abundance of 24 nt siRNAs relative to 21 nt siRNAs can be explained by the relative ratio of DCL3 to DCL4 dicing activities in plant tissues (Tabara et al. 2018). Moreover, the activity of DCL4 is post-translationally regulated in response to the cellular redox state (Seta et al. 2017; Tabara et al. 2018). This view is also supported by the *dcl* mutant analysis of *Physcomitrium*, as the *DCL4* knockout mutant Δ*Ppdcl4* shows severe developmental abnormalities; however, such a Δ*Ppdcl4* phenotype can be fully suppressed by the additional introduction of a *PpDCL3* knockout mutation (Arif et al. 2012). This indicates that the activity ratio of DCL3 to DCL4, i.e., which type of siRNAs are produced from the precursor RNAs, is also critical to development in mosses. It is important to note that, in *Arabidopsis*, the occurrence of PTGS or TGS depends on the downstream AGO-guided steps (changes in the ratio AGO1-siRNA target cleavage, an example of PTGS, versus AGO4-siRNA-guided DNA methylation, an example of TGS) (Wierzbicki et al. 2008, 2009). It is highly probable that a similar dependence might be present in bryophytes. In other words, during land plant evolution, plants would have acquired a fine-tuning system for growth and development by balancing PTGS and TGS according to environmental situations.

Interestingly, AtDCL2 and AtDCL4 have redundant functions: to produce viral- and transgene-derived siRNAs (Blevins et al. 2006; Deleris et al. 2006; Fusaro et al. 2006; Henderson et al. 2006). Recently, it has also been suggested that suppression of AtDCL4 activity promotes the biosynthesis of 22 nt siRNAs by AtDCL2, which, in turn, triggers a stress response (Wu et al. 2020). Thus, AtDCL2 and AtDCL4 are key DCLs for the regulation of plant growth and morphogenesis in response to the environment. However, comprehensive genomic analyses revealed that the *DCL2* family genes can be found only in seed plants (Axtell et al. 2007; Huang et al. 2015; Bowman et al. 2017; Belanger et al. 2023), suggesting that the DCL2-derived 22 nt siRNAs could have emerged in the seed plant lineage (You et al. 2017; Belanger et al. 2023). Additional groups of DCL proteins, such as monocot-specific DCL5 (formerly DCL3b), have also emerged during seed plant evolution, increasing the complexity of the small RNA system in plants (Patel et al. 2021).

In the *Physcomitrium* genome, there are four genes encoding canonical *DCL* proteins: *PpDCL1a* and *PpDCL1b*, which are most closely related to *AtDCL1*, and *PpDCL3* and *PpDCL4*, which are most closely related to *AtDCL3* and *AtDCL4*, respectively (Axtell et al. 2007). In addition, a specific DCL protein MINIMAL DICER-LIKE (mDCL) lacking an N-terminal helicase and a Dicer dimerization domain has been identified in addition to canonical types of PpDCL proteins (Coruh et al. 2015). All *Physcomitrium* mutants of canonical *DCL* genes are defective for developmental processes; the juvenile-to-adult transition, in particular, is accelerated in the Δ*Ppdcl3* mutant (Cho et al. 2008). A similar phenotype was shown in the *mdcl* mutant, indicating that the mDCL protein, like PpDCL3, also contributes to the hc-siRNA pathway. Interestingly, the *mdcl* mutation uniquely affected the accumulation of 23 nt and 24 nt siRNAs: the 23 nt and 24 nt siRNAs were increased and decreased, respectively, in *mdcl* mutants (Coruh et al. 2015), indicating *Physcomitrium*-specific mechanistic complexity of hc-siRNA biogenesis. In addition to such species-specific aspects of DCL proteins, the *Physcomitrium* DCL data have also revealed the evolutionarily conserved system of PpDCL1 autoregulation (Arif et al. 2022). The intron region of *DCL1* genes in *Arabidopsis* and *Physcomitrium* encodes miRNAs, which are distinct in sequence and position in the two species. Thus, the processing of intronic miRNAs and pre-mRNA splicing of host *DCL1* would compete mechanistically with each other. Since DCL1 functions in pri-miRNA processing, intronic miRNA biogenesis depends on the level of DCL1, resulting in the autoregulation of DCL1 levels (Rajagopalan et al. 2006; Axtell et al. 2007). Arif et al. (2022) demonstrated increased accumulation of the intronic miRNA miR1047 during abiotic stress and abscisic acid (ABA) treatment in *Physcomitrium*, but there was no increase in the *PpDCL1* transcript itself. The deletion mutant Δ*miR1047* showed hypersensitivity to abiotic stress (Arif et al. 2022). Additionally, *Arabidopsis* miR838 hosted in an *AtDCL1* intron was also upregulated during heat stress (Baev et al. 2014). Therefore, these data suggest the importance of conserved DCL1 autoregulation for stress tolerance in land plants.

**Fig. 2:**
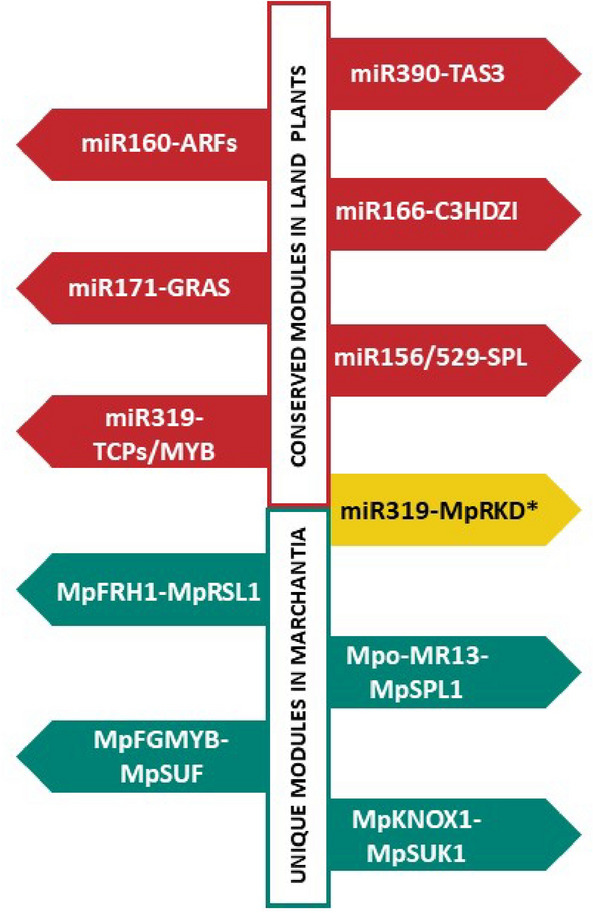
Graphical representation of miRNA–target modules conserved across three plant species, *M. polymorpha, P. patens* and *A. thaliana.* The modules conserved among all land plants are shown in red, and modules unique to *Marchantia* are shown in green. One of the unique modules, miR319–Mp*RKD*, consists of conserved miRNA, but the target is *Marchantia* specific; therefore, it is shown distinctly in yellow (Tsuzuki et al. 2016, 2019; Flores-Sandoval et al. 2018; Lin and Bowman 2018; Hisanaga et al. 2019a; Thamm et al. 2020; Dierschke et al. 2021; Csicsely et al. 2023; Futagami et al. 2023; Streubel et al. 2023; Yelina et al. 2023)

The *Marchantia* genome encodes four DCL genes: Mp*DCL1a*, Mp*DCL1b*, Mp*DCL3* and Mp*DCL4* (Lin et al. 2016; Lin and Bowman 2018; Pietrykowska et al. 2022; Belanger et al. 2023; Figs. 1 and 2). The MpDCL1a, MpDCL3 and MpDCL4 proteins contain basically the same functional domains as *Arabidopsis*, suggesting the functional conservation of these MpDCL proteins for small RNA biogenesis (Pietrykowska et al. 2022; Csicsely et al. 2023). Recently, Csicsely et al. (2023) reported the phenotype of Mp*dcl*^*ge*^ mutants (genome-edited Mp*dcl* mutants) generated by the CRISPR/Cas9 system. This study revealed severe developmental defects in the Mp*dcl1a*^*ge*^ mutant, whereas no detectable abnormalities were found in Mp*dcl1b*^*ge*^ or Mp*dcl4*^*ge*^ mutants, apart from the less frequently produced and smaller antheridiophores (Fig. 1). Importantly, the obtained Mp*dcl1a*^*ge*^ mutant plant had two amino acid substitutions and two amino acid insertions within the genomic region encoding the RES III domain, which probably affects the secondary protein structure, impairing the endonucleolytic activity of the MpDCL1a protein. The authors could not find large genomic rearrangements or early stop codons within the Mp*DCL1a* locus. All these data indicate that MpDCL1a has a dominant function in small RNA production in *Marchantia* (Fig. 1) and that its complete knockout is lethal, as in other plant species. Interestingly, the Mp*dcl1b*^*ge*^ mutant showed salt tolerance, while the three other kinds of Mp*dcl*^*ge*^ mutants displayed ABA hypersensitivity. Moreover, Mp*dcl3*^*ge*^ showed 1-naphthaleneacetic acid insensitivity for thallus growth inhibition and rooting enhancement (Csicsely et al. 2023). These phenotypes of Mp*dcl*^*ge*^ mutants strongly suggest the functional diversity of MpDCL proteins during *Marchantia* development, such as the involvement of MpDCL1b and MpDCL3 in small RNA biogenesis for salt tolerance and auxin signaling, respectively.

Recent comprehensive genomic analysis of plant DCL proteins demonstrated that the origin of DCL1 family proteins can be traced back to green algae, whereas the DCL3 and DCL4 families would have been acquired after the terrestrialization of plants (Belanger et al. 2023). Thus, the plant small RNA system is hypothesized to have evolved in the following order: the miRNA pathway (by DCL1) then the hc-siRNA pathway (by DCL3) and 21 nt tasiRNA/phasiRNA (phased secondary small interfering RNAs) pathways. Consistent with this suggestion, Tsuzuki et al. (2016) showed that the miR390–*TAS3* regulatory module is conserved in *Marchantia* (Fig. 2). Additionally, the proportion of siRNAs within total small RNAs is lower in *Marchantia* vegetative tissues than in *Arabidopsis*, possibly reflecting the relatively simple DCL system (i.e., lacking DCL2) in this liverwort. Further profiling of small RNAs in Mp*dcl*^*ge*^ mutants can provide additional clues of the mechanistic aspects of DCL proteins in *Marchantia*, leading to further insights into the evolution of the small RNA regulatory system for plant development.

## Role of selected small RNA and long non-coding RNA modules in plant development

As mentioned in the introduction, regulatory RNA species participate in many processes related to plant growth and development. For example, in *Marchantia*, miRNA–mRNA target modules, as well as long ncRNAs, influence thallus development, phase transitions and sexual/asexual reproduction. In this chapter, we review and compare reports on ncRNA regulatory modules in *Marchantia*, *Physcomitrium* and *Arabidopsis*. Figure 2 summarizes sRNA and lncRNA modules described to date that are unique to *Marchantia* and those that are conserved among these three plant species.

### Conserved miRNA–target modules across land plants

#### The miR390–*TAS3* module is involved in the regulation of ARF clade B transcription factor functions

Auxins are involved in many aspects of plant growth and development and in plant defensive responses to various stresses (Ludwig-Muller 2011; Balzan et al. 2014; Gomes and Scortecci 2021). AUXIN RESPONSIVE FACTORS (ARFs) represent a family of transcription factors (TFs) that regulate the expression of downstream auxin-regulated genes (Liu et al. 1993; Liscum and Reed 2002; Chapman and Estelle 2009). There are three evolutionary lineages of *ARF* genes in land plants: clades A, B and C. ARF transcription activators (*ARF5*, *ARF6*, *ARF7*, *ARF8* and *ARF19* in *Arabidopsis*) are all clustered in the A clade, whereas ARF transcription repressors are split into the B and C clades (Finet and Jaillais 2012). In *Arabidopsis*, *ARF* genes of clade B (*ARF2*, *ARF3* and *ARF4*) are preferentially targeted by trans-acting ARF-targeting small interfering RNAs (*TAS3*-derived tasiARFs) (Finet and Jaillais 2012; Xia et al. 2017). *Arabidopsis* miR390 triggers the production of tasiRNAs from the *TAS3* locus, which, in turn, target and downregulate mRNA levels of *ARF3* and *ARF4* (Montgomery et al. 2008; Marin et al. 2010; Rubio-Somoza and Weigel 2011; Endo et al. 2013). Furthermore, ARF3 and ARF4 activity promotes the juvenile-to-adult vegetative phase transition. ARF3 is required for floral meristem determinacy, and its gene is a direct target of APETALA 2 (AP2), which represses *ARF3* expression (Liu et al. 2014). Thus, AP2 and miR390 prolong the juvenile phase by repressing ARF3 and/or ARF4 activity (Fahlgren et al. 2006; Garcia et al. 2006; Rubio-Somoza and Weigel 2011). In *Arabidopsis*, miR390 is mis-paired exactly at the cleavage site of the *TAS3* RNA 5′ target site, which results in a non-cleavable target that is critical for *TAS3*-derived tasiRNA biogenesis.

In contrast, miR390 at the *TAS3* RNA 5′ target site is perfectly matched in bryophytes (Axtell et al. 2006; Xia et al. 2017; Lin and Bowman 2018). As in *Arabidopsis*, *TAS3* genes in bryophytes generate tasiRNAs that target *ARF* class B mRNA; however, they also generate tasiRNAs that target mRNA of *AP2* orthologs. In *Physcomitrium*, the miR390–*TAS3* module acts in the gametophytic stages of development to modulate gametophore initiation, protonemal branch determinacy and caulonemal differentiation (Plavskin et al. 2016). *TAS3* genes in *Physcomitrium* include two miR390 target sites and generate two tasiRNAs: tasiAP2 and tasiARF (Axtell et al. 2007; Krasnikova et al. 2013). It is known that, in *Physcomitrium*, the overexpression of miR390 results in slower gametophore formation, increased miR390-triggered tasiRNA accumulation and a decrease in the number of tasiRNA targets (Cho et al. 2012).

In *Marchantia*, there is one *TAS3* gene that contains two miR390 target sites (Krasnikova et al. 2013). Mp*TAS3* RNA generates tasiAP2, which regulates Mp*AP2* expression. However, whether a functional *TAS3–ARF* module is present in *Marchantia* is not clear. Generally, in plants, tasiARFs target *ARF* mRNA regions that encode a highly conserved amino acid sequence: K/RVLQGQE (Xia et al. 2017). Interestingly, a similar sequence is present in the MpARF2 protein but not in MpARF3. *Arabidopsis* ARF3 and *Marchantia* ARF2 (but not MpARF3) belong to the same clade, clade B (Finet and Jaillais 2012; Xia et al. 2017). Consequently, there is no evidence that tasiARF targets Mp*ARF3* mRNA, but an expected cleavage site has been found for Mp*ARF2* mRNA (Lin and Bowman 2018). Despite the presence of *TAS3*, *ARF* clade B and *AP2* genes and miR390 in *Marchantia*, there still is no report indicating the involvement of these elements in the juvenile-to-adult phase transition in this liverwort.

In summary, the miR390–*TAS3* module regulates the juvenile-to-adult phase transition in *Arabidopsis* and directs proper developmental timing in *Physcomitrium*. Meanwhile, the exact functions of the miR390–*TAS3* module in *Marchantia* still need to be determined (Fig. 1, left lower side).

#### The miR160–*ARFs*s module is involved in the regulation of ARF clade C transcription factor functions

In angiosperms, the *MIR160* family is involved in the regulation of class C *ARF* genes, which have been implicated in various biological processes during plant development. In *Arabidopsis*, miR160 downregulates *ARF10*, *ARF16* and *ARF17* gene expression (Rhoades et al. 2002; Mallory et al. 2005; Li et al. 2016). It was discovered that, in *Arabidopsis*, a 3′ regulatory region located downstream of the *MIR160a* gene contains auxin-response elements that enhance *MIR160* gene expression and upregulate miR160 levels (Liu et al. 2010b). *Floral organs in carpels* (*foc*) *Arabidopsis* mutant plants have a transposon insertion in the 3′ regulatory region of *MIR160.* This insertion results in reduced expression of *MIR160* and upregulated expression of *ARF10*, *ARF16* and *ARF17* genes in flowers. The *foc* mutant displays defects in floral organ formation, such as reduced fertility, the appearance of floral organs inside siliques and irregular flower shape, as well as aberrant seeds and viviparous seedlings (Liu et al. 2010b). Defects observed in the *foc* mutant are the result of an aberrant response to auxin since ARF10, ARF16 and ARF17 are involved in auxin signaling. Plants expressing miR160-resistant *ARF17* (35 S:5m*ARF17* lines) with increased *ARF17* mRNA levels display male sterility (Shi et al. 2015). A proper ratio of the miR160–*ARF* module components also has a significant impact on vegetative development. *Arabidopsis* plants expressing miR160-resistant *ARF17* displayed reduced plant size, leaf shape defects and reduced lateral root number (Mallory et al. 2005). Therefore, miR160-guided post-transcriptional gene regulation plays a pivotal role in the realization of successful vegetative and generative developmental programs.

In *Physcomitrium*, mRNAs of *ARF*s belonging to class C are also regulated by miR160 (Plavskin and Timmermans 2012; Plavskin et al. 2016). However, there is a lack of functional studies on the *Physcomitrium* miR160–*ARF* module. Similarly, in *Marchantia*, miR160 regulates the expression of Mp*ARF3*, a class C *ARF* (Lin et al. 2016; Tsuzuki et al. 2016). Mp*ARF3* is a negative regulator of cell proliferation and differentiation and inhibits the developmental transition from asexual to sexual reproduction (Flores-Sandoval et al. 2018; Tsuzuki et al. 2019). Pleiotropic defects caused by both loss-of-function and gain-of-function mutations in Mp*ARF3* were visible throughout the gametophytic stage of the life cycle. Loss-of-function Mp*arf3* alleles exhibited several developmental effects, including smaller cells, more distinct air pores, more pegged rhizoids, more gametophores per thallus area, ectopic antheridia and fused archegoniophores. Mutants overexpressing Mp*ARF3* exhibited improper thallus shape, with a lack of air chambers and pores. Likewise, Mp*mir160* knockout mutants were characterized by improper differentiation of the thallus, including air pores, and also of the gemma cups and gametophores (Fig. 1, left upper side) (Flores‐Sandoval et al. 2018b). In summary, miR160–*ARF*s derived from clade C modules are evolutionarily conserved between bryophytes and angiosperms. In both cases, these modules act as crucial players for the coordination of both the vegetative and reproductive phases of development.

#### The miR166–*C3HDZ1* (*HD-ZIPIII*) module regulates plant architecture

In angiosperms, the *MIR*165/166 family contains numerous members, which have diverged in function and specificity (Coruh et al. 2015; Merelo et al. 2016; Yan et al. 2016; Y. Li et al. 2022). *Arabidopsis* miR165/166 regulates the expression of the cognate C3HDZ TF gene family (HD-ZIPIII) members: *REVOLUTA* (*REV*), *PHABULOSA* (*PHB*), *PHAVOLUTA* (*PHV*) and *ARABIDOPSIS THALIANA HOMEOBOX 8* and *15* (*ATHB8*, *ATHB15*) (McConnell et al. 2001; Otsuga et al. 2001; Tang et al. 2003; Kim et al. 2005). These modules regulate transitions during organ development (Rhoades et al. 2002; Floyd and Bowman 2004; Tsuzuki et al. 2016; Yip et al. 2016; Ma et al. 2020). They are known to regulate adaxial/abaxial leaf polarity, shoot meristem maintenance, root meristem size and growth, vascular patterning and flower development (McConnell et al. 2001; Juarez and Timmermans 2004; Kidner and Martienssen 2004; Leibfried et al. 2005; Williams et al. 2005; Husbands et al. 2009; Sakaguchi and Watanabe 2012; Singh et al. 2014; Tsuzuki et al. 2016). Mutations that disrupt the pairing of *Arabidopsis REV*, *PHB* or *PHV* mRNAs with miR165/166 have similar effects on leaf phenotype, including reduced leaves with adaxial characteristics all around the circumference of the leaves (McConnell and Barton 1998; Mallory et al. 2004). These observations imply that related HD-ZIPIII TFs (REV/PHB/PHV) regulated by miR165/166 play similar roles in leaf morphogenesis.

In *Physcomitrium*, there are six *MIR166* genes (Kozomara et al. 2019). Ectopic expression of miR166, which targets *HD-ZIPIII* mRNAs, causes shorter stature of gametophores and altered leaflet shape and size (Yip et al. 2016). In *Marchantia*, the miR165/166 family is represented by two members: miR166a and miR166b (Tsuzuki et al. 2016). miR166a regulates Mp*C3HDZ1* mRNA levels (Lin et al. 2016; Tsuzuki et al. 2016; Lin and Bowman 2018). Mp*C3HDZ1* is upregulated in archegoniophores and antheridiophores compared to *Marchantia* vegetative thalli (Bowman et al. 2017). Furthermore, ectopic overexpression of the Mp*MIR166a* gene resulted in unregulated dorsoventral morphogenesis of the vegetative thallus (ventral curling) and downregulation of Mp*C3HDZ1* expression. Moreover, gemma cup structure was strongly reduced while gemmae were generated (Fig. 1, middle right side) (Tsuzuki et al. 2016).

Although the *Marchantia* thallus, *Physcomitrium* leaflets and *Arabidopsis* leaves represent different types of organs with unrelated origins (the *Marchantia* thallus and *Physcomitrium* leaflets represent gametophytes, while leaves represent a part of the *Arabidopsis* sporophyte), their proper polarity is optimal to perform the functions adapted to each plant’s requirements and is governed by the evolutionarily conserved miR165/166–*HD-ZIPIII* module.

#### The miR171–*GRAS* module controls plant growth during the vegetative phase of life

The *MIR171* family is considered one of the oldest miRNA families and was probably already present in the common ancestor of all embryophytes (Han and Zhou 2022). miR171 regulates the expression of members of the *GRAS* TF gene superfamily. GRAS is named after the first three GRAS proteins discovered in *Arabidopsis*: GIBBERELLIC ACID INSENSITIVE (GAI), REPRESSOR OF GA (RGA) and SCARECROW (SCR) (Di Laurenzio et al. 1996; Peng et al. 1997; Silverstone et al. 1998). In *Arabidopsis*, miR171 specifically targets three *HAIRY MERISTEM* (*HAM1*–*3*) genes belonging to the GRAS superfamily. These genes are also called *LOST MERISTEM* (*LOM*) or *SCARECROW-LIKE* (*SCL*) genes (Han and Zhou 2022). Specifically, miR171 downregulates the expression of several SCL TFs: SCL6/SCL6-IV, SCL22/SCL6-III and SCL27/SCL6-II (Ma et al. 2014). *HAM* family genes are required for the maintenance of indeterminate growth in angiosperms. *Arabidopsis ham* mutants exhibit a remarkably unique phenotype of shoot meristem and differentiation arrest (Stuurman et al. 2002; Engstrom 2012). In the case of *SCL* genes, it was also shown that they are involved in chlorophyll biosynthesis in *Arabidopsis* (Bolle 2004; M. H. Lee et al. 2008). Plants overexpressing miR171-resistant *SCL27* turn yellow and show a significant decrease in chlorophyll content. In contrast, plants overexpressing *MIR171c* and *scl6 scl22 scl27* triple mutants produce dark green leaves, with 40% more chlorophyll than wild-type plants (Ma et al. 2014). These results indicate that, in *Arabidopsis*, miR171-targeted SCLs are negative regulators of chlorophyll biosynthesis.

In *Marchantia*, Mp*SCARECROW-LIKE (*Mp*GRAS10*/Mp*SCL)* mRNA has been shown to be a target of miR171 (Lin et al. 2016; Lin and Bowman 2018; Yelina et al. 2023). However, neither the Mp*scl* nor the Mp*mir171* mutants showed any observable changes in chlorophyll accumulation. Thus, it seems unlikely that the miR171–Mp*SCL* module controls chlorophyll biosynthesis. However, some effects on thallus morphology in Mp*scl* mutants, such as a stunted thallus with inward-curling edges, were observed (Fig. 1, lower right side) (Yelina et al. 2023). In *Physcomitrium*, *PpGRAS12* is a validated target for miR171 (Axtell et al. 2007; Hiss et al. 2017). Deletion of the *PpGRAS12* gene has a negative effect on sporophyte production, as fewer sporophytes were formed in *ppgras12* knockout plants compared to wild-type plants. Additionally, at the protonema stage, highly selective and unique growth arrest was seen in plants with inducible *PpGRAS12* overexpression (Beheshti et al. 2021). Although *GRAS* family members regulated by miR171 play important roles in shoot meristem differentiation and protonema proliferation, respectively, in *Arabidopsis* and *Physcomitrium*, more functional studies are required to determine the role of the *Marchantia* miR171–Mp*SCL* module.

#### The miR156/529–*SPL* module is involved in the vegetative-to-reproductive phase transition

It has been shown that *Arabidopsis* miR156(a-h) targets transcripts of several *SQUAMOSA PROMOTER BINDING PROTEIN-LIKE* TF mRNAs (*SPL2*/*3*/*4*/*5*/*9*/*10*/*11*/*13*/*15*) and regulates the timing of flowering (Wang et al. 2009; Wang 2014; Wang and Wang 2015; Zheng et al. 2019; Jerome Jeyakumar et al. 2020). *Marchantia* miR529 shares 17 of the 21 nucleotides of *Arabidopsis* miR156 and could be considered an miRNA of the same family. Additionally, computational analyses found that miR529c has a complementary sequence to Mp*SPL2* mRNA in a similar context to the *Arabidopsis* miR156–*SPL* mRNA module. It was hypothesized that miR529c targets the *SPL* gene mRNA in *Marchantia* and might have a conserved/common biological function with *Arabidopsis* although liverwort will not set flowers. Once Mp*mir529c*-null mutants (Mp*mir529c*^*ge*^ mutants) were established with the use of the CRISPR/Cas9 system, surprisingly, the mutant thalli immediately started to transform into sexual-like reproductive organs (gametangia) and produced gametes. This development was observed even in the absence of far-red light and long-day conditions, both of which are applied normally to induce reproductive development on the bench. This result suggested that loss of miR529c function caused gametangium formation and gamete production without the environmental changes normally needed for the reproductive transition (Fig. 1, upper group). Next, an miR529c-resistant Mp*SPL2* transgene (mMp*SPL2* gene) was established and introduced into wild-type plants. The transgene producing miR529c-resistant mRNA induced a reproductive transition in the whole area of thalli without far-red light conditions, as was seen in Mp*mir529c*^*ge*^ mutants. The Mp*SPL2* mRNA levels were elevated in both Mp*mir529c*^*ge*^ and mMp*SPL2* mutant plants. It was thus suggested that the transition to reproductive development in liverworts is controlled by the miR156/529–*SPL* module in a similar manner to angiosperms, even though reproductive processes differ greatly between angiosperms and liverworts (Tsuzuki et al. 2019).

Interestingly, in the moss *P. patens*, both miR156 and miR529 are present. However, they differ in expression patterns, with miR156 mainly expressed in the protonema and miR529 mainly expressed in gametophores with developed sporophytes (Xie et al. 2021). Of 13 members of the *Physcomitrium SPL* family, only transcripts of three *SPL* genes are proven targets of miR156. These three moss miR156-targeted *SPL*s are in the same phylogenetic group as the miR156-targeted *SPL* genes in *Arabidopsis* (Riese et al. 2007, 2008; Streubel et al. 2023). Functional studies indicated that miR156 promotes the vegetative developmental transition from the protonema to the leafy gametophore in *P. patens*, while Pp*SPL3* acts in the opposite direction (Cho et al. 2012). The role of miR529 in sporophytes is still unknown.

The miR156/529–*SPL* module influences developmental transitions in the vegetative phase of the life cycle in both bryophytes and flowering plants. Interestingly, the directionality of miR156/529 action is conserved between liverworts and angiosperms, as the miRNAs repress the vegetative-to-reproductive phase transition. In moss, miR156 promotes the vegetative transition from young filamentous gametophytic tissue to the adult leafy shoot form.

#### The miR319–*TCP*s/*MYB*/Mp*RKD* module governs vegetative and generative developmental pathways

In angiosperms, the conserved miR319/159 family is involved in various processes, such as leaf development, hormone signaling, organ curvature and cell proliferation (Palatnik et al. 2003; Allen et al. 2007; Rubio-Somoza et al. 2014; Schommer et al. 2014). Nearly half of *TEOSINTE BRANCHED*/*CYCLOIDEA*/*PCF* (*TCP*) mRNAs and *R2R3-MYB* TF mRNAs are targeted by miR319 in *Arabidopsis*. It has been accepted that miR159 and miR319 are both essential for the regulation of the developmental process in *Arabidopsis*. The *jaw-D* mutation was first identified by genetic screening in *Arabidopsis*; the mutant plants have serrated rosette leaves, epinastic cotyledons and crinkled leaves (Palatnik et al. 2003). At that time, it was revealed that miR319 is coded in the *JAW* locus and targets the mRNAs of some TCP TFs. The *jaw*-D mutant phenotype was caused by the overexpression of miR319a (Palatnik et al. 2003). In addition, mutations in the *MIR319a* and *MIR319b* genes caused a moderate reduction in the size of leaf serrations, while the *MIR319a*/*b* mutation almost completely suppressed their formation. This suggests that both these genes play a mostly quantitative role in the formation of leaf serrations (Koyama et al. 2017). Moreover, miR319 regulates senescence and jasmonate biosynthesis in *Arabidopsis* by targeting *JAZ* genes. It was shown that overexpression of miR319 delayed leaf senescence and reduced jasmonate levels, while inhibition of miR319 accelerated leaf senescence and increased jasmonate levels (Schommer et al. 2008). Based on several studies, it is generally accepted that *Arabidopsis MIR319* and *MIR159* were derived from a common ancestor miRNA gene, and they are often regarded as constituting one miRNA family due to high sequence similarity and common non-canonical processing steps from the loop-to-base direction before maturation (Palatnik et al. 2007; Bologna et al. 2009; Li et al. 2011). Based on degradome data, loop-to-base processing of pri-miR319 was also reported in the moss *Physcomitrium* (Addo-Quaye et al. 2009). Moreover, *MIR159* is absent in mosses.

In *Physcomitrium*, miR319 is known to control the levels of *MYB* gene expression. Although there is a low degree of complementarity between miR319 and the *MYB* gene, experiments have shown that miR319 can guide the cleavage of the *MYB* mRNA (Xia et al. 2016). The intricate and multifaceted biological function of miR319, as well as relatively lesser-known functions of this miRNA in liverworts, encouraged research on *Marchantia*. *Marchantia* plants overexpressing *MIR319b* showed abnormal and obvious morphological phenotypes in thallus and gemma cup structure. It was demonstrated that thalli in 35 S:Mp*MIR319b* plants were curled to the dorsal side. Additionally, this ectopic expression triggered the absence of visible gemma cups while gemmae were generated (Tsuzuki et al. 2016). Moreover, Mp*mir319a*^*ge*^ and Mp*mir319b*^*ge*^ null mutants were established using the CRISPR/Cas9 system. They showed normal thallus development, which was intriguing, but they could produce a smaller number of gemmae and gemma cups on thalli with sizes similar to those of the wild type. Interestingly, Mp*mir319a*/*b*^*ge*^ double knockout plants showed even fewer gemma cups and gemmae than single mutants. Importantly, double mutant plants produced sexual reproductive organs (gametangia), but at a much-reduced frequency compared to wild-type plants. Additionally, these male and female gametangia showed irregular shapes, possibly reflecting asymmetric organ formation during their development (Futagami et al. 2023). RNA degradome analysis found a complementary sequence to miR319 in Mp*RKD* (*RWP-RK DOMAIN-CONTAINING*) mRNA and a cleavage site (Lin et al. 2016). The complementary sequences are localized just downstream of the termination codon of the Mp*RKD* mRNA. It was previously reported that Mp*RKD* is preferentially expressed in the egg and sperm cells and that it is involved in germ cell formation (Koi et al. 2016). In addition, Mp*rkd* mutants were described to show slightly asynchronous thallus growth and lacked outgrowths at the edges of gemma cups, although gemmae were produced. To determine whether the observed defects in *mir319a* and *b* mutants were caused by unregulated expression of the *RKD* gene, an miR319-resistant *RKD* gene was constructed under the direction of the *RKD* gene promoter and introduced into a wild-type liverwort. Plants expressing the resistant *RKD* gene showed no gemma cups or gemmae and phenocopied *mir319a*, *mir319b* and *mir319a*/*b* mutants quite well (Futagami et al. 2023). *Marchantia* has two *TCP* and 22 *R2R3-MYB* genes. Of these, a complementary sequence to miR319 could be found only in the Mp*MYB21* gene. RNA degradome and 5′ RACE analysis detected *R2R3-MYB21* mRNA cleavage (Lin et al. 2016; Tsuzuki et al. 2016). As in the *RKD* gene, the mir319-resistant form of the *MYB21* gene was established and placed under the direction of the *MYB21* promoter then introduced into liverwort to obtain transgenic lines. The transgenic plants grew thalli of smaller size with gemma cups/gemmae at a similar density. The phenotype was almost identical to that of transgenic liverwort plants to which mir319-sensitive *MYB21* was similarly introduced. The available data suggested that miR319a or miR319b downregulates Mp*RKD* function, possibly in a spatial manner, and induces the pathway for gemma cup/gemma formation (Futagami et al. 2023). The implications of the presented findings are vast, as miR319 has been shown to regulate complex and diverse developmental pathways in plants, including *Arabidopsis*, *Physcomitrium* and *Marchantia*. As such, further investigation of the biological function of miR319 is crucial for a better general understanding of plant growth and development.

### *Marchantia*-specific non-coding RNA–target modules

#### The MpFRH1–Mp*RSL1* module is involved in the development of various thallus structures

MpFRH1–Mp*RSL1* is an example of a unique module identified only in *Marchantia* so far. *RSL* (*ROOT HAIR DEFECTIVE SIX-LIKE 1*) class 1 genes encoding basic helix–loop–helix TFs are known to positively regulate the development of root hairs in vascular plants and rhizoids in liverwort and moss, respectively (Proust et al. 2016; Honkanen et al. 2018; Thamm et al. 2020). In *Marchantia*, a liverwort-specific miRNA called FEW RHIZOIDS1 (MpFRH1; also called Mp-miR11861; Tsuzuki et al. 2016; Thamm et al. 2020) was found to negatively regulate a single *RSL* class 1 gene, Mp*RSL1*. In contrast, no FRH1 recognition sequence was detected in the *RSL* class 1 transcripts in the moss *P. patens*, the lycophyte *Selaginella kraussiana* or the angiosperm representative *A. thaliana* (Honkanen et al. 2018). Moreover, FRH1 miRNA was not found in the moss, lycophyte or angiosperm representatives. Hence, the FRH1 miRNA is thought to have originated only in the liverwort lineage. It is possible that this miRNA is also present in other liverwort species since a conserved target site has been identified in orthologs of the Mp*RSL1* gene in representatives from the classes Marchantiopsida and Jungermanniopsida (Honkanen et al. 2018). Mp*frh1* loss-of-function plants showed more rhizoid cell groups than wild-type plants. A similar phenotype of more groups of rhizoid cells was observed in Mp*rsl1* gain-of-function transgenic plants. Moreover, higher levels of Mp*RSL1* mRNA were present in Mp*frh1* loss-of-function mutants (Thamm et al. 2020). Hence, MpFRH1 miRNA was shown to act as a repressor of rhizoid cell development in *Marchantia*. Additionally, Mp*frh1* gain-of-function and Mp*rsl1* loss-of-function transgenic plants had no rhizoids or rarely developed rhizoids, produced empty gemma cups or were characterized by lower production of gemmae and lacked multicellular papillae. Therefore, it seems that Mp*RSL1* positively regulates the development of *Marchantia* thallus structures derived from a single epidermal cell: gemmae, rhizoids and papillae (Fig. 1, bottom left first group) (Honkanen et al. 2018; Thamm et al. 2020).

Although the function of *RSL* class 1 genes has been shown to be conserved, the mechanisms that regulate their expression differ between liverworts and angiosperms. In *Arabidopsis*, the GLABRA2 (GL2) TF negatively regulates *RSL* class 1 genes. The AtGL2 protein directly interacts with L1 box motifs present in the promoters of two *RSL* class 1 genes, At*RHD6* and At*RSL1* (Lin et al. 2015). Through this interaction, the AtGL2 protein inhibits the initiation of root hair development by suppressing the transcription of *RSL* class 1 genes. However, no miRNA–*RSL1* modules have been identified thus far in angiosperms or mosses. Therefore, distinct land plant lineages have developed diverse and independent negative regulators to govern the expression of conserved genes responsible for the root or rhizoid development essential for terrestrial life.

#### The Mpo-MR-13–Mp*SPL1* module is involved in meristem dormancy control

*SPL* genes are found in all green plants, ranging from unicellular green algae to angiosperms, where they act as important regulators of diverse developmental processes (Chen et al. 2010; Preston and Hileman 2013; Xu et al. 2016). In angiosperms, many members of the *SPL* gene family are post-transcriptionally regulated by miR156/157, miR529 and miR535 (Xu et al. 2016; Li et al. 2022a; Tregear et al. 2022; Zhang et al. 2022). miR156/157–*SPL* regulatory pathways have been found to be conserved in most land plants. In *Arabidopsis*, 10 of 16 *SPL* genes have been found to be targets of miR156/157 (Wang et al. 2009; Preston and Hileman 2013; Xu et al. 2016). In *M. polymorpha*, four *SPL* genes have been identified: Mp*SPL1*–Mp*SPL4* (Tsuzuki et al. 2016, 2019; Bowman et al. 2017; Streubel et al. 2023). Mpo-MR-13 (also called Mp-miR11671) was identified as a unique miRNA-targeting Mp*SPL1* gene in *Marchantia* (Lin et al. 2016; Tsuzuki et al. 2016; Streubel et al. 2023). Mpo-MR-13 might be a liverwort-specific miRNA, as its target sites were found to be conserved in *SPL* sequences of Jungermanniopsida and Marchantiopsida representatives, respectively (Streubel et al. 2023).

Phylogenetic studies have shown that, of the *Arabidopsis SPL* family, At*SPL8* is the closest relative of Mp*SPL1* (Flores-Sandoval et al. 2018; Streubel et al. 2023). However, At*SPL8* mRNA is not regulated by miR156. In *Arabidopsis*, At*SPL8*, along with miR156-targeted *SPLs*, regulates anther development (Unte et al. 2003). However, there are no functional studies showing the involvement of Mp*SPL1* in reproductive organ development in *Marchantia*. Meristem dormancy is a feature of the shade-avoidance response observed in angiosperms, which results in repressing the outgrowth of lateral meristems. To investigate the possible roles of the Mpo-MR-13–Mp*SPL1* module in the shade-avoidance response in *Marchantia*, several mutant plants were generated. Mp*spl1* loss-of-function plants showed a more branched phenotype than wild-type plants and no dormant meristems. On the contrary, Mp*spl* gain-of-function plants developed fewer active meristems and, consequently, had increased dormant meristems compared to wild-type plants. As expected, Mpo-*mr-13* loss-of-function plants showed a similar phenotype to Mp*spl1* gain-of-function plants, i.e., more dormant meristems (Streubel et al. 2023). Hence, the Mpo-MR-13–Mp*SPL1* module was shown to be important for meristem dormancy and branching during the shade-avoidance response in the thallus of *Marchantia* (Fig. 1, upper group). Additionally, this study demonstrated that the Mpo-MR-13–Mp*SPL1* module is controlled by PIF-mediated phytochrome signaling (Streubel et al. 2023). A similar dependence was proposed in *Arabidopsis*, in which PIF-mediated repression of the expression of several *MIR156* genes releases the miR156-targeted *SPLs* from post-transcriptional control, enabling them to pursue the shade-avoidance mechanism (Xie et al. 2017). However, which At*SPL* gene is involved in shade avoidance is still unknown. Therefore, distinct miRNAs but similar mechanisms of *SPL*-regulated meristem dormancy important for the shade-avoidance mechanism evolved independently in the liverwort and angiosperm lineages.

#### The Mp*FGMYB*–Mp*SUF* lncRNA module governs sex determination in *Marchantia*

Like 70% of other liverwort species, sex in *Marchantia* is determined by the sex chromosomes U (female) and V (male) (Renner et al. 2017; Hisanaga et al. 2019b). The U-chromosome has a “Feminizer” locus, which has been shown to be dominant, since plants possessing both U and V chromosomes ultimately develop female sexual organs. This U-linked gene from the Feminizer locus, named Mp*BPCU*, is a member of the plant-specific Basic Pentacysteine (BPC)/Barley B Recombinant (BBR) TF family. MpBPCU was shown to be necessary for archegoniophore development, as Mp*bpcu* mutants do not produce sex organs. A genomic fragment containing the Mp*BPCU* gene was sufficient to convert from a male to a female phenotype (archegoniophore development) when introduced into the background of male individuals; however, egg cells could not develop because of the lack of other U-linked genes essential for the differentiation of egg cells. An Mp*BPCU* gametolog, Mp*BPCV*, was found on the male chromosome. *BPCU* and *BPCV* genes function in reproductive phase induction in females and males, respectively, but only *BPCU* was reported to be the master sex determinant for female sex determination in *Marchantia* (Iwasaki et al. 2021). The Feminizer, an MpBPCU TF, was shown to regulate the autosomal sex-determining locus, Mp*FGMYB*–*SUF*. The Mp*FGMYB*–*SUF* module consists of the *FEMALE GAMETOPHYTE MYB* (Mp*FGMYB*) TF gene and *SUF* (*SUPPRESSOR OF FEMINIZATION*), which encodes an lncRNA. Mp*FGMYB* encodes an R2R3-MYB–type TF specifically expressed in archegoniophores to promote female sexual program progression. *SUF* is transcribed in an antisense direction from the promoter located at the 3′ end of the Mp*FGMYB* locus. *SUF* is expressed mainly in antheridiophores, but it is also present in the male vegetative thalli. Expression of the *SUF* gene inhibits transcription of the Mp*FGMYB* gene, thereby suppressing the female gametogenesis program in males. In females, the MpBPCU TF negatively regulates *SUF* expression by binding to GAGA motifs enriched in the *SUF* promoter region. Consequently, *SUF* is not transcribed, resulting in the successful expression of Mp*FGMYB*, which further promotes feminization in plants (Hisanaga et al. 2019a) (Fig. 1, upper right side).

Comparing the genetic elements involved in sexual organ development in *Physcomitrium* and *Marchantia*, it is important to note that sex chromosomes are not present in *P. patens.* However, two homologs of Mp*FGMYB* (Pp3c6_23360 and Pp3c5_7650) were found to be highly expressed in moss archegonia (Pu et al. 2020). Whether these two homologs are essential for female sexual organ development in mosses is not clear. On the other hand, in *Arabidopsis*, there are seven orthologs of MpFGMYB, of which three proteins, namely, MYB64, MYB98 and MYB119, have been reported to function in female gametogenesis (Hisanaga et al. 2019a, 2019b). At*MYB98* has been shown to be expressed specifically in synergid cells and is essential for their differentiation (Dubos et al. 2010). At*MYB64* and At*MYB119* are expressed transiently during embryo sac development and are especially crucial for embryo sac cellularization. At*MYB119* expression was shown to be regulated by histidine kinase CYTOKININ-INDEPENDENT 1 (CKI1). *CKI1* expression was found mainly in the central cell nucleus and, to a lesser extent, in the egg cell nucleus (Pischke et al. 2002; Hejatko et al. 2003). CKI1 activity is necessary for the specification of the developmental program of the central cell and antipodal cells, with concomitant suppression of egg cell and synergid cell program development in central and antipodal cells (Yuan et al. 2016). Therefore, the role of FGMYBs in female gametophyte differentiation in *Marchantia* and *Arabidopsis* is retained, but their regulatory pathways are different.

#### The Mp*KNOX1*–Mp*SUK1* lncRNA module is critical for sporophyte development

The eukaryotic life cycle alternates between the haploid and diploid phases. In phylogenetically diverse eukaryotes, paralogous homeodomain (HD) protein families are implicated in the haploid-to-diploid reprogramming of gene expression upon fertilization (Herskowitz 1989; Zhao et al. 2001; J. H. Lee et al. 2008; Hedgethorne et al. 2017). In the green plant lineage, they are represented by the KNOTTED1-like homeobox (KNOX) and BEL1-like homeodomain (BELL) protein families. Importantly, to fulfill their functions, *KNOX* and *BELL* genes need to be expressed in gametes to enable KNOX/BELL protein heterodimerization just after gamete fusion (Bowman et al. 2016). This HD protein system is also functional in *Marchantia.* Notably, gametic expression of class I Mp*KNOX1* and Mp*BELL2*/*3*/*4* genes is necessary for diploid sporophyte development, as revealed by the analysis of loss-of-function mutant plants. In the case of the Mp*KNOX1* gene, a similar mode of expression regulation by lncRNA was identified, as in the case of the Mp*FGMYB*–Mp*SUF* module. The Mp*KNOX1* gene is expressed specifically in mature egg cells, and, following fertilization, its expression is observed during sporophyte development until the future sporogenous cells become distinct. Interestingly, an antisense lncRNA *SUPPRESSOR OF KNOX1* (Mp*SUK1*) is transcribed from an intergenic promoter and overlaps with the last two exons of the Mp*KNOX1* locus. Unlike the expression profile observed for Mp*KNOX1*, Mp*SUK1* expression is predominantly observed in antheridia, suggesting that this lncRNA may act in *cis* to repress Mp*KNOX1* expression during antheridium development (Fig. 1, upper right side) (Dierschke et al. 2021). Although similar expression activation of the three *Physcomitrium* class I *KNOX* genes (*MKN2, MKN4* and *MKN5*) was observed, an lncRNA regulatory module was not found in any of these genes (Singer and Ashton 2007; Sakakibara et al. 2008). Instead, polycomb-mediated repressive chromatin modification is required to maintain gametophyte identity through repression of the sporophyte genetic program (Mosquna et al. 2009; Okano et al. 2009). In the case of triple loss-of-function of class I *KNOX* moss mutant plants (MKN2-4-5dis-1), sporophytes exhibited defects in cell proliferation during development (Singer and Ashton 2007; Sakakibara et al. 2008). Therefore, in both bryophytes, the class I *KNOX* genes retained the ancestral function of controlling the haploid-to-diploid transition, but the sporophytic expression pattern suggests their neofunctionalization as stimulators of cell proliferation in the diploid generation.

Further, during land plant evolution, class I *KNOX* genes were confined to functioning in the maintenance of sporophytic meristematic cells. In *Arabidopsis*, all four members of the class I *KNOX* family participate in a different developmental process throughout the sporophytic life phase. However, their expression in specific domains of SAM preserves the pool of indeterminate meristematic cells and specifies the boundaries for the propagation of lateral organs and the stem during plant development (Hay and Tsiantis 2010). For example, *SHOOT MERISTEMLESS* (*STM*) is the first class I *KNOX* gene expressed during early embryogenesis, and its constant expression is critical for shoot meristem formation and maintenance (Barton and Poethig 1993; Long et al. 1996; Scofield et al. 2007, 2008). Additionally, *STM* plays an essential role in the transient activity of the central floral stem cells and subsequently directs the formation of carpels and the placental tissues from which ovules arise (Long et al. 1996; Long and Barton 2000). The boundaries between class I *KNOX-*expressing and non-expressing cells need to be precisely established, which requires the repression of *KNOX* gene expression in developing organs. This repression is achieved by a complex regulatory network, in which proteins like ASYMMETRIC LEAVES1 and 2 (AS1 and AS2) bind directly to the *KNOX1* gene promoters to repress their transcription. Also, the combined actions of two polycomb repressive complexes (PRCs) suppress class I *KNOX* transcription by chromatin modifications (Byrne et al. 2000; Ori et al. 2000; Xu et al. 2003; Katz et al. 2004; Guo et al. 2008; Xu and Shen 2008).

The available data on class I *KNOX* genes demonstrate that, in the representatives of different land plant branches, class I *KNOX* genes are crucial factors controlling the proliferation status of the diploid generation. Although class I *KNOX* genes have the same function in the studied bryophytes, the mechanisms that regulate their expression are different. In fact, the *Physcomitrium KNOX* gene repression mechanism using the PRC complex resembles the one of *Arabidopsis*, while in *Marchantia* the lncRNA Mp*SUK1* is the major regulator of the Mp*KNOX1* gene expression profile.

## Coordination among non-coding RNA regulatory modules drives *Marchantia polymorpha* development

Despite the substantial heterogeneity in growth conditions and specialized multicellular body forms, it was recently reported that there is significant conservation of transcription programs across most organs in vascular plants. In the case of the *Physcomitrium* and *Marchantia* transcriptomes of leaf-like organs (leaflets and thallus, respectively), similarities but also prominent differences were observed when compared with the transcriptomes of vascular plants (Julca et al. 2021). Additionally, the specificity of regulatory programs is also visible in plant microtranscriptome compositions; for example, in *Marchantia*, of 265 miRNAs only seven are conserved with those of other land plants (Tsuzuki et al. 2016; Bowman et al. 2017; Pietrykowska et al. 2022).

**Fig. 3:**
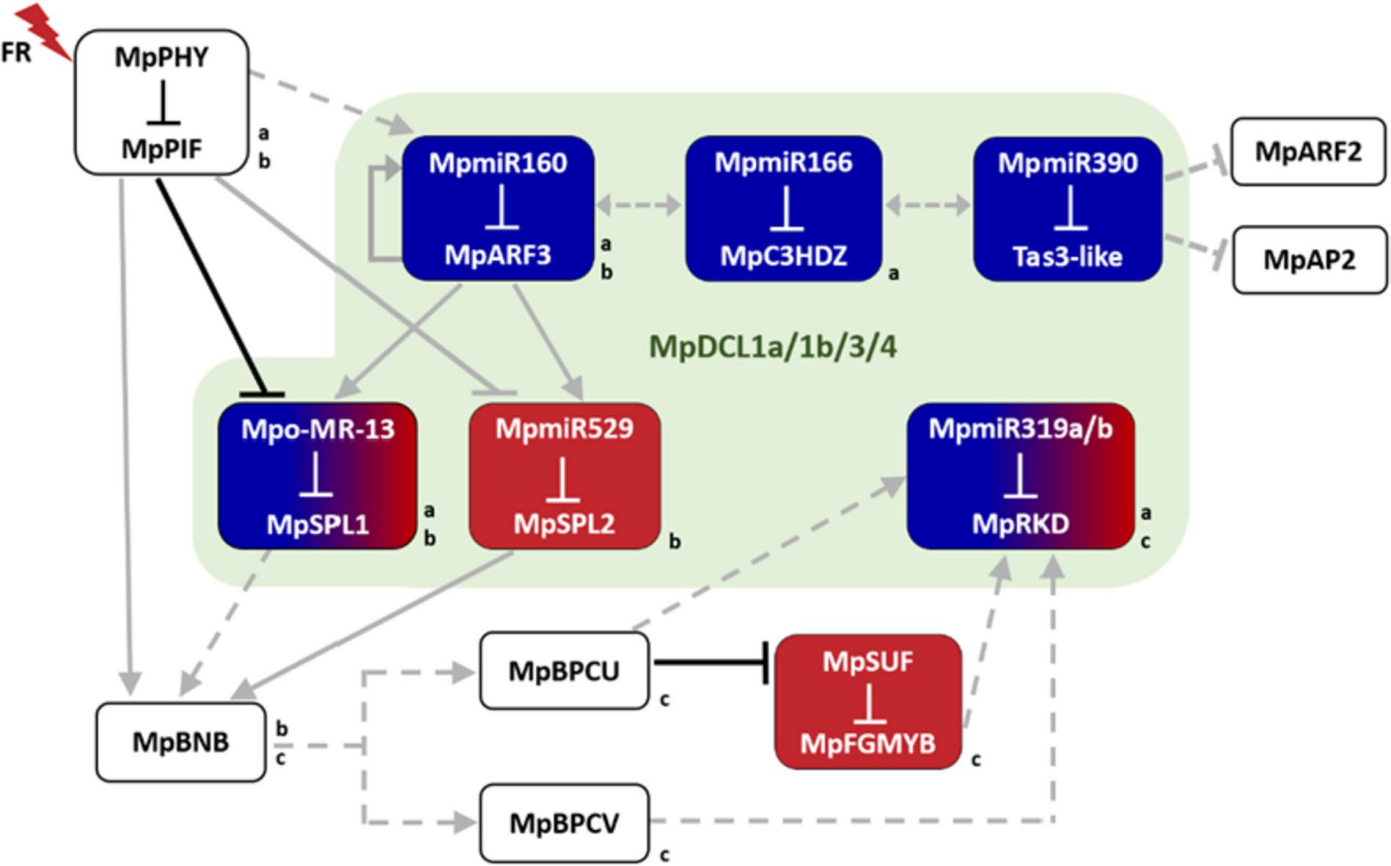
Putative new connections between regulatory pathways involved in vegetative and reproductive development in *M. polymorpha*. Colored boxes indicate experimentally confirmed non-coding RNA–target modules. Modules shown in blue and red boxes are involved in vegetative and reproductive development, respectively, whereas modules shown in a blue to red gradient are involved in both vegetative and reproductive development. Black bars indicate regulatory pathways that have been experimentally validated, gray arrows and bars indicate regulatory pathways suggested in the cited papers and dashed gray arrows and bars indicate regulatory pathways suggested in this review. The lowercase letters on the right sides of boxes indicate whether the gene or module is involved in the vegetative developmental program **(a**), the vegetative-to-reproductive phase transition **(b)** or the reproductive developmental program **(c)**

The gathered data indicate that ncRNAs play crucial roles in controlling various developmental processes during the *M. polymorpha* life cycle. By targeting major TFs, *Marchantia* small RNAs coordinate the balance between plant growth and development. However, to properly fulfill their molecular functions, small RNAs must be precisely processed by multiprotein complexes. Mutations in machineries responsible for their biogenesis most often have pleiotropic effects on plant phenotypic features, as has been reported in *Arabidopsis* and many other plants (Jacobsen et al. 1999; Golden et al. 2002; Liu et al. 2005; Xie et al. 2005; Khraiwesh et al. 2010; Parent et al. 2015). A similar effect was also observed in *Marchantia*, when each of four *DCL* genes present in the *Marchantia* genome was mutated (Csicsely et al. 2023). It has been suggested that MpDCL1a is responsible for miRNA biogenesis since mutants of this gene showed the most severe abnormalities during development. Changes in antheridiophore morphology in Mp*dcl1b*^*ge*^ and Mp*dcl4*^*ge*^ mutant plants and the lack of antheridiophore production in Mp*dcl3*^*ge*^ suggest that these three proteins may be involved in the processing of a specific group of small RNAs that are important for the regulation of male reproductive branch formation. Therefore, the cooperative action of all four DCL proteins generates an interwoven network of miRNA and siRNA regulatory pathways that are necessary for successful vegetative and reproductive developmental programs in *Marchantia* (Fig. 3).

The most profound and robust impact on the *Marchantia* life cycle is exerted by the miR160–*ARF* clade C module, as nearly all aspects of growth and development were affected when Mp*ARF3* or Mp*MIR160* activity was disturbed (Flores-Sandoval et al. 2018b). This result suggests that the Mp*ARF3* gene is a superior factor in coordinating the cell proliferation and differentiation processes, resulting in maintaining an accurate balance between the meristematic and differentiated states in a cell or tissue. Interestingly, it was also proposed that Mp*ARF3* forms a negative feedback loop with miR160 to regulate its own expression level. In Mp*arf3* mutant plants, the Mp*MIR160* transcript was significantly downregulated, while, in plants expressing an additional copy of the Mp*ARF3* gene under its native promoter, the miR160 level was upregulated compared to in wild-type plants. Therefore, Mp*MIR160* transcription appears to depend on MpARF3 activity, suggesting that MpARF3 activates its own repressor (Fig. 3) (Flores‐Sandoval et al. 2018b). Gene co-expression analysis in different tissues and developmental stages of *Marchantia* revealed that Mp*ARF3* forms a co-expression group with several genes that are related to the vegetative-to-reproductive phase transition and are highly active in reproductive structures. Among similarly expressed genes, three TFs were recognized: Mp*C3HDZ*, Mp*SPL1* and Mp*SPL2.* Mp*C3HDZ* and Mp*SPL2* are post-transcriptionally regulated by conserved miR166 and miR529c, respectively, and Mp*SPL1* is targeted by Mpo-MR-13, which is unique to *Marchantia* (Flores-Sandoval et al. 2018a). Although the reported TF expression dynamics were similar to those of Mp*ARF3*, analysis of the transcriptomes of gain- and loss-of-function mutants of the miR160–Mp*ARF3* regulatory module, along with mutant plant phenotype analysis, revealed that MpARF3 in fact inhibits the reproductive transition in *M. polymorpha.* MpARF3 activates the expression of Mp-*MR-13* and Mp-*MIR529c* genes to downregulate mRNA levels of Mp*SPL1* and Mp*SPL2*, respectively, resulting in prolonged vegetative growth (Flores-Sandoval et al. 2018a; Flores‐Sandoval 2018b). The relationship between Mp*ARF3* and the miR166–Mp*C3HDZ* module is not clear, as no additional analyses are available. From *Arabidopsis* studies, it is known that the HD-ZIPIII–miR165/166 signaling pathway, among others, is essential for normal SAM functions (Williams et al. 2005). *Marchantia* plants overexpressing miR166a revealed deregulated dorsoventral morphogenesis of the thallus, which might be the consequence of Mp*C3HDZ* downregulation. Therefore, a properly balanced interplay of the miR160–Mp*ARF3* and miR166–Mp*C3HDZ* modules might be required for the precise coordination of cell proliferation and differentiation processes in the *Marchantia* thallus (Fig. 3).

As described in the previous section, the miR529c–Mp*SPL2* module was shown to control the vegetative-to-reproductive phase transition in *Marchantia* (Tsuzuki et al. 2019). In contrast, the Mpo-MR-13–Mp*SPL1* module is involved in the shade-avoidance mechanism. This module depends on the red/far-red light signaling pathway, with the Mp*PHY*–Mp*PIF* module promoting Mp*SPL1* activity by repressing Mpo-*MR-13* gene transcription. Downregulation of Mpo-MR-13 results in meristem dormancy (Streubel et al. 2023). This phytochrome-dependent far-red high irradiance signaling also induces the sexual reproduction program (Inoue et al. 2019), and, as shown by Streubel et al. (2023), it promotes the expression of the Mp*SPL2* gene (Streubel et al. 2023). However, whether it acts through Mp*MIR529c* repression or an increase in transcription of the Mp*SPL2* gene was not specified. According to transcriptomic data, the expression of Mp*SPL1*, like that of Mp*SPL2*, is upregulated in gametangiophores (Bowman et al. 2017; Flores-Sandoval et al. 2018). Therefore, besides its role in meristem dormancy in shade conditions, Mp*SPL1* might be an important factor for *Marchantia* sexual reproduction. These data raise the intriguing possibility that the Mp*SPL1* gene might play a dual function in *Marchantia* development. Upon phytochrome signaling during shade conditions, Mp*SPL1* first activates the meristem dormancy program. Second, it might enable the cellular program to switch to the reproductive program, which could be cooperatively orchestrated by both MpSPL1 and MpSPL2 TFs. Collectively, based on the available data, regulatory pathways involved in the vegetative-to-reproductive phase transition in *Marchantia* can be proposed (Fig. 3). The Mp*PHY*–Mp*PIF* module acts as a master regulator to control the activation of genes related to sexual reproduction. Among those, the expression of two *Marchantia SPL* family members is promoted through transcriptional repression of two miRNA genes, Mpo-*MR-13* and Mp*MIR529c*. Under low far-red light conditions, Mpo-MR-13 and miR529c, along with the MpARF3 TF, inhibit the reproductive program, so they prolong vegetative growth. As miR160 downregulates Mp*ARF3* expression, it promotes the vegetative-to-reproductive phase transition and thus acts in concordance with the phytochrome signaling pathway. An interesting question arises: do the Mp*PHY*–Mp*PIF* and miR160–Mp*ARF3* modules act separately, or does one of them influence the other? There is no information concerning the influence of a high far-red light regime on Mp*MIR160* or Mp*ARF3* expression. However, similar to the Mpo-*MR-13* gene promoter, in the promoter region of Mp*MIR160*, there are two PBE-box motifs. The PBE-box motif is a regulatory sequence that could potentially be recognized by the MpPIF protein (Xie et al. 2017; Streubel et al. 2023). Although it has been proposed that MpPIF mediates the repression of Mpo-*MR-13* expression by recognition of the PBE-box motif in its promoter region, MpPIF could selectively promote Mp*MIR160* expression, as was already shown for other *Marchantia* genes (Inoue et al. 2019).

Another layer of germline determination within the *Marchantia* vegetative thallus is Mp*PHY*–Mp*PIF*–mediated expression activation of the basic helix-loop-helix (bHLH) TF Mp*BONOBO* (Mp*BNB*), an autosomal gene (Yamaoka et al. 2018). Constitutive Mp*BNB* expression, like constitutive Mp*SPL2* expression, was sufficient to induce gametangium and gametangiophore production, irrespective of light conditions. However, in contrast to Mp*SPL2*, the lack of which caused only a delay in the development of sexual structures, knockout of Mp*BNB* completely suppressed gametangium and gametangiophore production. These observations suggest that Mp*BNB* is a master regulator of gametangial progenitor cell programming, whereas Mp*SPL2* stimulates the induction of a reproductive program, probably in cooperation with other factors, for example, Mp*SPL1*. Both these genes have similar accumulation profiles, i.e., both are upregulated in the meristematic region (Yamaoka et al. 2018; Tsuzuki et al. 2019). Interestingly, within the 5 kb promoter region of the Mp*BNB* gene, four SBP-responsive elements can be found (SBP–SPL response element). Therefore, the miR156/529–Mp*SPL2* module may be one of a few coordinately working units assisting the phytochrome-mediated activation of Mp*BNB* expression (Fig. 3). Although Mp*BNB* expression is restricted to gametangium initial cells, this activation is a prerequisite for further gametangium development and sexual branch formation. How Mp*BNB* influences further germ cell differentiation and gametophore development is not clear, and the mechanism is still unknown. Similar effects on sexual organ formation show Mp*BPCU* and Mp*BPCV* gametologs, which are essential for reproductive phase induction in females and males, respectively. However, unlike Mp*BNB*, Mp*BPCU* and Mp*BPCV* are expressed already in seven-day-old female and male thalli (Iwasaki et al. 2021). Although they are present during the vegetative phase of growth, they are not sufficient to trigger a switch from the vegetative program to the reproductive program. The data showing that Mp*BNB* is a master gene for reproductive program initiation suggest that the MpBNB protein may be important for MpBPCU and MpBPCV protein activation so that they can work together to direct gametangiophore and gamete development. It is also possible that some other undiscovered factor(s) induced by far-red light might be important for the activity of the MpBPCU and MpBPCV proteins, leading to gametogenesis (Fig. 3). Importantly, the U-chromosome–localized *BPCU* gene is a feminizer locus that, by repression of the *SUF* lncRNA, enables *FGMYB* gene expression, which further initiates female program continuation. *BPCV* has no effect on the *FGMYB–SUF* locus but is important for proper male program continuation (Fig. 3). Although recently it was shown that the miR319–Mp*RKD* module plays a role in proper gemma/gemma cup development (Futagami et al. 2023), an earlier study described Mp*RKD* as a crucial player for the final steps of egg and sperm cell development (Koi et al. 2016; Rovekamp et al. 2016). However, the effect of miR319 during reproductive organ development is unclear, as its expression profile was not examined in parallel with Mp*RKD* expression. Additionally, though Mp*RKD* acts downstream of the Mp*BPCU*/*V* gametolog and Mp*FGMYB*–*SUF* module, whether one of these TFs promotes Mp*RKD* expression is also unknown (Fig. 3). Therefore, investigation of functional relationships among genes engaged in the successful progression of each step during the reproductive phase of the *Marchantia* life cycle is needed.

Although several conserved miRNA–target modules were identified in *Marchantia*, some of them still lack detailed functional studies, for example the miR390–*TAS3* module. In angiosperms, this module controls the expression levels of clade B ARF members. Although small RNA analysis revealed the presence of tasiARFs that could target the Mp*ARF2* gene and tasiAP2, which could regulate members of the Mp*AP2* family (Fig. 3), the role of the miR390–*TAS3* module during the *Marchantia* life cycle is still unresolved. In angiosperms, the miR390–*TAS3*–*ARF* module is indispensable for the regulation of auxin signaling and for leaf morphology, among other functions (Chitwood et al. 2009; Marin et al. 2010; Skopelitis et al. 2012). As described earlier, the adaxial-abaxial cell fate program is controlled by miR165/166–*HD-ZIPIII*, and ARF3/ARF4 TFs are regulated by tasiARFs (McConnell et al. 2001; Otsuga et al. 2001; Emery et al. 2003; Chitwood et al. 2009; Skopelitis et al. 2012). Together, these interactions exactly define and preserve the boundaries between the adaxial and abaxial sides of the leaf. As both regulatory modules, miR166–*HD-ZIPIII* and miR390–*TAS3*–*ARF*, are present in the *M. polymorpha* genome, an interesting question arises: are there similar dependencies of the functions of these two modules in the *Marchantia* thallus as observed during angiosperm leaf development? The thallus clearly shows characteristic dorsiventrality, with zonation of the upper assimilatory region, forming air chambers and gemma cups, the middle storage region with parenchymal cells and the ventral epidermis with scales and rhizoids (Shimamura 2016). Therefore, detailed functional studies are needed to investigate whether these two conserved small RNA modules may act in concert to establish each region’s boundaries and whether they influence one another (Fig. 3).

Since the discovery of thousands of lncRNAs based on genome-wide analysis of plant transcriptomic data, the functional relevance of these RNAs has been demonstrated in only a few cases (Chen et al. 2020; Wierzbicki et al. 2021). Within the *Marchantia* transcriptome, almost 30,000 transcripts were described as undefined due to the lack of similarity to currently available plant and animal genomic data. It was suggested that some of these transcripts might be portions of long UTRs or encode lncRNAs (Lin et al. 2016). *SUF* is an example of such a functional lncRNA expressed exclusively in male individuals to suppress the expression of Mp*FGMYB* (Hisanaga et al. 2019a). A similar mode of action was observed in the case of Mp*SUK1* lncRNA, which represses Mp*KNOX1* expression in antheridia (Dierschke et al. 2021). Both Mp*SUF* and Mp*SUK1* are involved in sexual reproduction (Fig. 3), which is the function of many lncRNAs observed across a range of eukaryotic species (Golicz et al. 2018). Currently, lncRNAs are emerging as potentially important players in various fundamental biological processes in plants, such as organ morphogenesis, tolerance to stress and response to pathogenic infection. Therefore, further research on lncRNAs in *Marchantia* is needed to learn what other processes, in addition to sexual reproduction, are supervised by lncRNAs. These studies will also increase our understanding of the mechanism of lncRNA function in non-seed plants and will indicate which aspects of lncRNA biology are universal and which are locus specific.

As we have shown, there are demonstrated and hypothetical interconnections among the ncRNA–target modules in different developmental processes during the *Marchantia* life cycle. Several are crucial for the successful progression through the vegetative phase of growth, while others are related to enabling the sexual reproduction program. However, our present understanding of these genetic networks represents only a skeletal framework, as many functional studies are still lacking. Therefore, future studies are needed to understand the biochemical functions of the yet uncharacterized module components, along with the manners in which the signaling pathways transmit the information that ultimately regulates the appropriate developmental stage.

## Data Availability

Enquiries about data availability should be directed to the authors.
